# An Efficient Method for Neuron‐Like Differentiation of SH‐SY5Y Neuroblastoma Cells Using Retinoic Acid and Laminin‐Rich Extracellular Matrix

**DOI:** 10.1002/dvg.70066

**Published:** 2026-08-02

**Authors:** Marina Mantellatto Grigoli, Bianca Cruz Pachane, Angelina Maria Fuzer, Sabrina Dorta de Oliveira, Ana Beatriz Aparecida Targas, Vanessa Alexandre‐Silva, Heloisa Sobreiro Selistre‐de‐Araujo, Patricia Regina Manzine, Marcia Regina Cominetti

**Affiliations:** ^1^ Department of Gerontology Federal University of São Carlos – UFSCar São Carlos Brazil; ^2^ Department of Physiological Sciences Federal University of São Carlos – UFSCar São Carlos São Paulo Brazil; ^3^ Molecular Oncology Research Center Barretos Cancer Hospital Barretos São Paulo Brazil; ^4^ Department of Public Health, Mental Health and Maternal‐Child Health, Faculty of Nursing University of Barcelona Barcelona Catalonia Spain

**Keywords:** extracellular matrix, laminin, neuronal differentiation, retinoic acid, SH‐SY5Y

## Abstract

The human neuroblastoma cell line SH‐SY5Y is widely used in vitro as a model due to its ability to acquire neuron‐like properties. Conventional differentiation protocols often rely on retinoic acid (RA), which can lead to limited stability of neuronal‐like phenotypes over time. In this study, we evaluated a differentiation approach combining RA with a laminin‐rich extracellular matrix (LrECM). This approach accelerated differentiation, with neuron‐like morphology and neurite outgrowth observed as early as 4 days and sustained features for up to 10 days. Immunofluorescence analysis indicated increased NeuN expression, and Western blot confirmed the sustained presence of β3‐tubulin during differentiation. Furthermore, a trend toward enhanced acetylcholinesterase (AChE) activity suggested partial cholinergic features. Altogether, these findings indicate that incorporating LrECM supports the stability of RA‐induced neuron‐like characteristics in SH‐SY5Y cells and may provide a useful alternative for studies of neuronal–phenotype differentiation.

## 1 Introduction

A commonly used in vitro approach involves differentiating the human neuroblastoma cell line SH‐SY5Y into cholinergic‐like neurons. This model is widely used in neuroscience research due to its ability to induce neuron‐like characteristics, defined here as neurite outgrowth, a polarized morphology, and expression of neuronal markers, such as β3‐tubulin and NeuN (de Medeiros et al. 2019; Xicoy et al. 2017). This process uses retinoic acid (RA) to induce differentiation by efficiently upregulating genes involved in synapse formation, maintenance, and function (Jahn et al. 2017; Pahlman et al. 1984). Henceforth, this represents a more cost‐effective and straightforward model than relying upon primary cultures (Feles et al. 2022), with the additional advantage of significantly reducing the time required for differentiation.

The absence of glial cells in a monoculture model results in a deficiency of endogenous neurotrophic factors, potentially leading to apoptosis. Consequently, supplementing the culture media with growth factors, including brain‐derived neurotrophic factor (BDNF), has become a standard practice to enhance their survival and differentiation (de Medeiros et al. 2019). The dependence on trophic factors presents potential issues, as their signaling can protect cells from neurotoxicity, thereby introducing a substantial challenge when studying cell death (Strother et al. 2021). Furthermore, the absence of an extracellular matrix (ECM) can significantly diminish the physiological relevance of this model, as it fails to provide essential mechanotransduction pathways required for proper adhesion and differentiation cues (Minegishi et al. 2023).

Laminins are a class of glycoproteins found in the ECM of the developing brain that promote neurite outgrowth in various neuronal cell types when studied in vitro (Powell and Kleinman 1997). They are critical adhesion molecules in hippocampal axon outgrowth, favoring stiffness‐dependent axon growth through the actin‐adhesion coupling (Minegishi et al. 2023). When cultured in a laminin‐rich matrix (LrECM), SH‐SY5Y expressed higher FAK levels and longer neurites compared to a fibronectin or collagen matrix, leading to morphological and biochemical differentiation (Dwane et al. 2013). In fact, laminin represents the most abundant ECM protein in the nervous system. It is essential for neural survival, migration, and differentiation, and several isoforms have been shown to promote neurite extension and neuronal maturation in vitro (Menezes et al. 2024; Mesquita et al. 2022; Chize et al. 2025). Due to the striking rise in neuronal differentiation and neurite outgrowth in an LrECM, we describe a refinement of the method to induce a faster, cost‐effective, and persistent differentiation of SH‐SY5Y using a combination of laminin and RA.

## 2 Materials and Methods

### 2.1 Cell Culture

SH‐SY5Y (CVCL_0019) human neuroblastoma cells were purchased from the Rio de Janeiro Cell Bank (BCRJ) and cultured in DMEM/F12 media (1:1, Gibco) supplemented with 10% heat‐inactivated fetal bovine serum (FBS, Vitrocell), 1% sodium pyruvate (Sigma‐Aldrich), and 0.1% non‐essential amino acids (Sigma‐Aldrich). Cells were maintained at 37°C in a saturated humidity environment containing 5% CO_2_ and were routinely sub‐cultured when confluency reached 80%–90%. For sub‐culturing and cell expansion, 2.1 × 10^6^ cells were seeded into T‐75 culture flasks, and passaging was performed every 48–72 h using trypsin (Vitrocell). Cultures were tested for *Mycoplasma* using conventional PCR and a luciferase‐based assay (MycoAlert, Lonza) prior to experimentation.

### 2.2 SH‐SY5Y Differentiation

SH‐SY5Y were differentiated into neuron‐like cells by treatment with all‐trans RA (Cayman Chemical Company) and LrECM. Undifferentiated control cells were maintained under standard culture conditions (DMEM/F12 supplemented with 10% FBS, 1% sodium pyruvate, and 0.1% non‐essential amino acids) without RA or LrECM coating. In addition, cells treated with RA (10 μM) in the absence of LrECM were included as an additional control to assess the individual effect of RA. Six‐well (35 mm) plate (Greiner bio‐one, Cat. No. 657160) bottoms were coated with ice‐cold Growth Factor Reduced (GFR)‐Matrigel (Corning, Ref. 354230) diluted in DMEM/F12 media (1:100). The composition of Matrigel, a commercially available matrix from the solubilized basement membrane of mouse Engelbreth‐Holm‐Swarm (EHS) sarcoma, used in this study included laminin (main component‐60%), collagen IV (30%), heparan sulfate proteoglycans, entactin/nidogen (8%), basic fibroblast growth factor (bFGF < 0.1 pg/mL), insulin‐like growth factor (IGF‐1, 5 ng/mL), transforming growth factor‐β (TGF‐β, 1.7 ng/mL), epidermal growth factor (EGF, < 0.5 ng/mL), platelet‐derived growth factor (PDGF, < 5 pg/mL), neuronal growth factor (NGF, < 0.2 ng/mL), and vascular endothelial growth factor (VEGF, 1.0‐1.5‐10 μg/mL, according to the manufacturer's specification; batch no. 13623001) was used for all experiments. After Matrigel solidification at 37°C for 30 min, SH‐SY5Y cells (2.5 × 10^5^ cells/well) were seeded and kept in DMEM/F‐12 advanced medium supplemented with 4 mM GlutaMXTM‐I (Gibco), 1% FBS, and RA (10 μM) for 48 h at 37°C, 5% CO_2_. Cells were maintained for up to 10 days with media replacement every 48 h.

### 2.3 Neurite Measurement

Neurite length was quantified using the FIJI software following a previously described method (Pemberton et al. 2018; Schindelin et al. 2012). After 4, 7, 9, and 10 days of differentiation, a minimum of five brightfield images were captured using a light microscope (Nikon Eclipse TS100‐F). Calibrated images were converted from RGB to 8‐bit, segmented using the “Find Maxima” tool, and converted into a binary image. Images were skeletonized and evaluated according to the macro described in the Data S1.

### 2.4 Immunofluorescence

SH‐SY5Y cells (3.75 × 10^4^ cells/cm^2^) were seeded in culture chamber slides and differentiated following the previously described protocol in the section *SH‐SY5Y differentiation*. After 4 or 9 days of differentiation, cells were fixed with a 4% paraformaldehyde (PFA) solution for 20 min and washed three times with 50 mM glycine‐PBS for 10 min each. Slides were blocked for 1 h, RT with immunofluorescence (IF) solution (1.3 M NaCl, 132 mM Na_2_HPO_4_, 34.5 mM NaH_2_PO_4_, 77 mM NaN_3_, 1% BSA, 2% Triton X‐100, and 0.5% Tween 20) supplemented with 10% BSA and pre‐absorbed goat‐derived antibody fragment (F(ab')2) (1:1000, Abcam Cat# ab98754). Samples were incubated overnight at 4°C with primary antibody for NeuN (1:250, Cell Signaling Technology Cat# 12943), washed thrice with IF solution for 20 min, and exposed to secondary antibodies conjugated with AlexaFluor 488 (1:500, Thermo Fisher Scientific Cat# A‐11034) and 568 (1:500, Molecular Probes Cat# A‐11011). Cells were counterstained with phalloidin‐A488 (Molecular Probes Cat# A12379) and DAPI (0.5 mg/mL, Abcam Cat# ab228549) before slide assembly using the Fluoromount G mounting media (EM Sciences Cat# 17984‐25). Cells were visualized under epifluorescence (ImageXpress Micro XLS, Molecular Devices) using 20× and 40× magnification. Imaging analysis was conducted using the FIJI software (Schindelin et al. 2012), and the macro pipeline is described in the Data S2.

### 2.5 Cell Lysate

Cell plates were placed on ice, and the supernatant was discarded. Cells were carefully washed with 1 mL PBS and removed using a cell scraper; this step was repeated three times. Cell suspensions were collected in 15 mL conical tubes on ice and spun at 152.9 g (1200 rpm) for 5 min at 4°C. The supernatant was discarded, and the cell pellet was resuspended in 100 μL of CelLytic lysis buffer (Sigma‐Aldrich) and transferred to a 1.5 mL microtube. The lysate was incubated at −80°C for 30 min and centrifuged at 15,294 g (12,000 rpm) for 15 min, 4°C. The supernatant was collected and stored frozen. Samples were collected on days 4 and 9 of the differentiation process. Total protein content was quantified using the BCA protein assay kit (Thermo Scientific) according to the manufacturer's instructions.

### 2.6 Western Blotting

Cell lysates (6.5 μg/μL) were diluted in 7 μL Laemmli buffer (Bio‐Rad) and boiled at 100°C for 5 min in a dry bath. Samples were added to 4%–20% Mini‐PROTEAN gradient gels (Bio‐Rad) with a protein ladder (Precision Plus Protein Dual Color Standards, Bio‐Rad) as a loading control, and electrophoresis was run at 100 V for 90 min. Proteins were transferred to nitrocellulose membranes using the Trans‐Blot Turbo Transfer System (Bio‐Rad) at 2.5 A and 25 V for 3 min. Transfer efficiency was confirmed by staining membranes with 0.2% Ponceau S solution in 1% acetic acid. Unspecific binding was blocked using Tris‐Buffered Saline (TBS) containing 0.5% Tween‐20 (TBST) with 5% casein milk solution for 1 h at RT. Membranes were successively washed with TBST and incubated overnight with a primary antibody for β3‐tubulin (Cell Signaling Technology Cat# 5666, 1:1000 in 5% BSA‐TBST). After three 5‐min washes with TBST, membranes were incubated with an antibody for β‐actin‐HRP (Cell Signaling Technology Cat# 5125; 1:1000 in TBS with 1% casein blocker) as an endogenous control for 1 h. Membranes were developed using the Clarity Western ECL substrate kit (Bio‐Rad) and captured using the ChemiDOC MP imaging system (Bio‐Rad). Band densitometry was performed using ImageJ. Digital images of membranes were acquired under non‐saturating conditions to ensure signal linearity. For each lane, regions of interest (ROIs) corresponding to the bands were manually defined, and the integrated density (area × mean gray value) was measured. The background signal was estimated from an adjacent area of the membrane with no visible bands and subtracted from each measurement. To account for loading variability, protein expression levels were normalized to the corresponding endogenous control (β‐actin) from the same lane. Relative protein expression was calculated as the ratio between the background‐corrected integrated density of the target protein and that of the endogenous control. All samples were analyzed in at least [insert number] independent experiments, and mean values were used for statistical analysis. Relative protein expression was obtained from the ratio between the mean pixel value of proteins of interest and the mean pixel value of the endogenous controls. Full‐length (uncropped) Western blot membranes are displayed in the Figure S1A–C.

### 2.7 Acetylcholinesterase Enzymatic Activity

Acetylcholinesterase (AChE, EC 3.1.1.7) activity was determined by a colorimetric assay kit (Elabscience). Cells were washed twice with PBS (pH 7.4), and total protein was extracted with a lysis buffer (20 mM HEPES, 150 mM NaCl, 1% NP40) supplemented with protease and phosphatase inhibitors (Sigma‐Aldrich). Sample absorbance was measured for 5 min at 412 nm using a BioTek Synergy H1 Hybrid Multi‐Mode Microplate Reader. Results were expressed in nmol/min/mg of protein based on the molar extinction coefficient of 1.98 × 10^4^.

### 2.8 Statistical Analysis

Data sets were checked for outliers using the ROUT method (Q = 0.1), and data distribution was determined by Shapiro–Wilk (*n* < 9) or D'Agostino‐Pearson omnibus K2 (*n* ≥ 9). All analyses were performed on both biological and technical replicates. All experiments were conducted on at least three independent occasions, each including two or three technical replicates. Parametric data sets were evaluated using one‐way ANOVA with Tukey's multiple comparison test. Nonparametric data sets were assessed using the Kruskal–Wallis analysis of variance with Dunn's multiple comparison test. Values of *p* < 0.05 were considered statistically significant. Statistical analysis and graph design were performed on GraphPad Prism (v. 10.4).

## 3 Results

### 3.1 Differentiation of SH‐SY5Y Cells Is More Efficient in Laminin‐Rich ECM Co‐Treatment With Retinoic Acid

**FIGURE 1:**
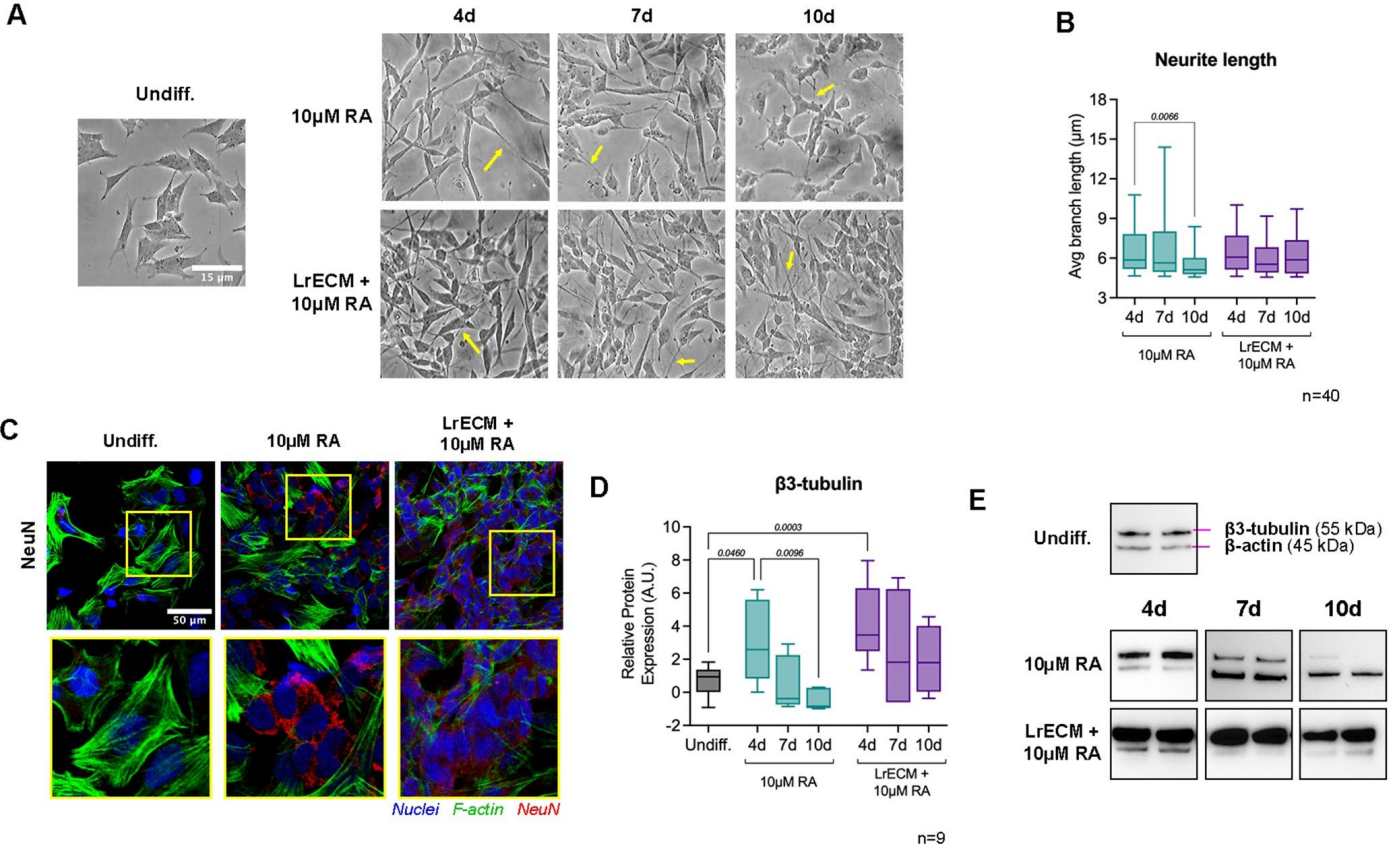
Characterization of SH‐SY5Y differentiation with laminin‐rich ECM and retinoic acid. (A) Representative brightfield images of SH‐SY5Y cells differentiated with retinoic acid (10 μM RA) or laminin‐rich ECM and retinoic acid (LrECM + 10 μM RA) for 4, 7, and 10 days. An undifferentiated control was maintained in parallel. Scale bar: 15 μm. (B) Measurement of neurite length in μm from differentiated cells. (C) Representative confocal fluorescence images of SH‐SY5Y cells stained with DAPI (nuclei, blue), phalloidin‐A488 (F‐Actin, green), and NeuN (red) after differentiation for 4 days with 10 μM RA or LrECM + 10 μM RA. Widefield images are shown above, and the zoomed details are shown below. Scale bar: 50 μm. (D) Densitometry of β3‐tubulin bands normalized by the β‐Actin control. Statistically relevant *p‐*values are displayed on top of comparison brackets. (E) Western blotting membranes were probed for β3‐tubulin (55 kDa) and β‐actin (45 kDa) as housekeeping control.

The differentiation of SH‐SY5Y with retinoic acid (RA) and laminin‐rich ECM (LrECM) was followed using light microscopy for up to 10 days. In this context, the standard 10 μM RA treatment was maintained in parallel. As expected, cells differentiated with 10 μM RA after 7 days exhibited a distinct phenotype, similar to neuronal morphology (Figure 1A). However, this phenotype was not fully maintained after 10 days, when the SH‐SY5Y cells began to de‐differentiate. The combination of LrECM and RA significantly enhanced neuronal differentiation in SH‐SY5Y cells compared to the traditional model, starting as early as 4 days and maintaining these characteristics for up to 10 days. Unlike the dedifferentiation observed with the RA‐exclusive protocol, this refined approach resulted in persistent neurite outgrowth and greater apparent surface coverage of the culture over time, reflecting neurite extension and improved cell attachment (Figure 1B; Figure S1D).

Neuronal markers NeuN and β3‐tubulin were also investigated to confirm our observations. Under qualitative analysis of immunofluorescence, NeuN was expressed in both the RA and LrECM+RA groups, albeit with a broader distribution in cells of the combined differentiation protocol after 4 days (Figure 1C). The protein expression of β3‐tubulin was examined using Western blotting, and corroborated our previous observations. There was a significant increase in β3‐tubulin after 4 days under both differentiation methods. However, while the RA group showed a steep decrease in β3‐tubulin expression after 10 days, the LrECM+RA group was able to maintain its levels for a longer period (Figure 1D,E). This suggests that the differentiation of SH‐SY5Y with LrECM and RA is more efficient than the RA‐exclusive protocol in maintaining neuronal characteristics in vitro.

### 3.2 Laminin‐Rich ECM Sustains Neuron‐Like Characteristics in SH‐SY5Y

**FIGURE 2:**
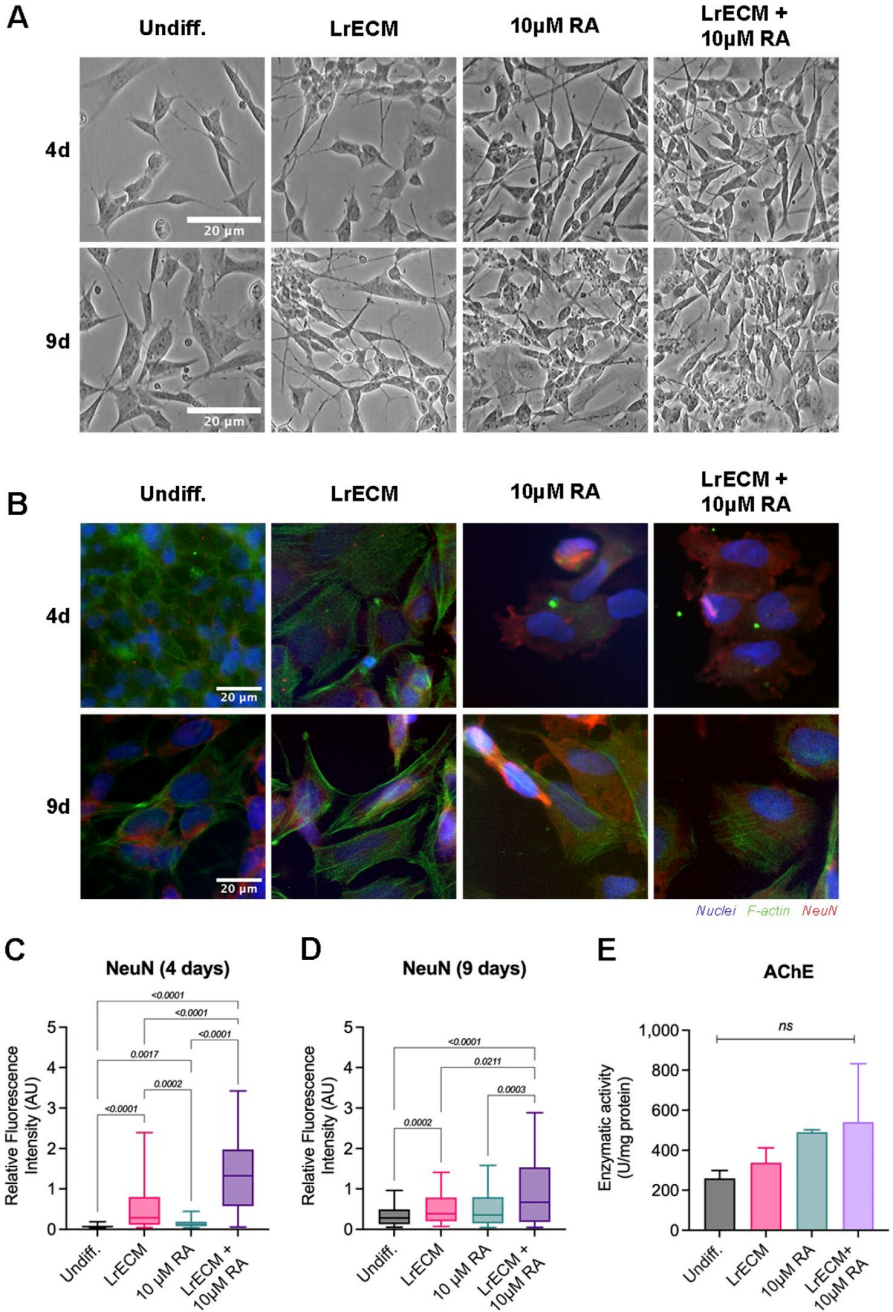
Laminin‐rich ECM helps to maintain retinoic acid‐derived differentiation. (A) Representative brightfield images of SH‐HY5Y cells differentiated with retinoic acid (10 μM RA) or laminin‐rich ECM and retinoic acid (LrECM + 10 μM RA) for 4 and 9 days. An undifferentiated control and an LrECM control were maintained in parallel. Images acquired under 40× magnification. Scale bar: 20 μm. (B) Representative epifluorescence images of SH‐SY5Y cells stained with DAPI (nuclei, blue), phalloidin‐A488 (F‐Actin, green), and NeuN (red) after differentiation for 4 and 9 days with 10 μM RA or LrECM + 10 μM RA, with LrECM and an undifferentiated control. Scale bar: 20 μm. (C,D) Quantification of the NeuN signal under epifluorescence after (C) 4 days and (D) 9 days. Statistically relevant *p‐*values are displayed on top of comparison brackets. (E) Enzymatic acetylcholinergic activity (AChE) of SH‐SY5Y cells after 4 days of differentiation with 10 μM RA and/or LrECM. ns = non‐significant (*p* ≥ 0.05).

To determine the impact of LrECM on SH‐SY5Y cell differentiation, we evaluated cell morphology and NeuN expression in both differentiation methods (RA and LrECM+RA). The LrECM promoted neurite development after 4 days and led to neuron‐like characteristics after 9 days. Still, this factor alone is not as proficient in differentiating cells as RA‐derived treatments (Figure 2A). The fluorescent imaging of NeuN indicated its expression in LrECM, RA and LrECM+RA groups, with a substantial upregulation in the combined RA and LrECM treatment and a qualitative change in NeuN distribution, with a tendency toward increased perinuclear localization after 9 days (Figure 2B). NeuN expression was quantitatively assessed (Figure 2C,D), whereas subcellular distribution was evaluated qualitatively. Interestingly, NeuN was more expressed in cells under LrECM than 10 μM RA after 4 days (Figure 2C). After 9 days of differentiation, both LrECM and LrECM+RA were able to sustain similar NeuN levels from the previous time point, suggesting its major contribution to the persistence of neuron‐like characteristics in SH‐SY5Y (Figure 2D).

We also assessed AChE enzyme activity expression after 4 days and found a non‐significant tendency of increased enzymatic activity in cells differentiated with 10 μM RA and LrECM (Figure 2E). Although this tendency may suggest an early modulation of cholinergic enzyme activity, it was not statistically significant under the evaluated conditions. Therefore, no conclusion can be drawn regarding enhanced cholinergic differentiation at this stage.

## 4 Discussion

The use of in vitro models provides a valuable means to study complex human diseases while minimizing ethical concerns. Among these, the SH‐SY5Y neuroblastoma cell line is widely applied due to its ability to acquire neuronal‐like properties under specific differentiation conditions (de Medeiros et al. 2019; Pemberton et al. 2018; Waetzig et al. 2019). RA remains the most common differentiation agent, while extracellular matrix (ECM)‐based approaches have also been explored to improve the neuronal‐like phenotype and stability (Dwane et al. 2013; Agholme et al. 2010; Shipley et al. 2016; Kaya et al. 2024).

In this study, we refined the RA‐induced differentiation protocol by incorporating a laminin‐rich extracellular matrix (LrECM) layer. Rather than accelerating neuronal maturation, this combination supported the maintenance of neuronal‐like morphology and marker expression for up to 10 days, indicating improved stability at later time points. Although early morphological changes were observed, functional specialization required prolonged culture, suggesting that LrECM stabilizes rather than enhances differentiation.

It is important to notice that this model does not aim to recapitulate fully mature neuronal networks. SH‐SY5Y‐derived cells are widely regarded as representing an early neuronal phenotype (Kovalevich et al. 2021). Indeed, recent work by Leuenberger et al. (Leuenberger et al. 2026) employed rigorous ultrastructural and functional analyses to demonstrate that SH‐SY5Y cells do not form morphologically or functionally complete synapses even under extended differentiation conditions, further establishing that these cells represent an early neuron‐like phenotype rather than synaptically mature neurons. Consistent with these findings, the present study did not evaluate synapse formation, synaptic transmission, or functional neuronal connectivity. The LrECM+RA protocol described here yields an early neuron‐like phenotype characterized by enhanced neurite outgrowth and stabilized neuronal marker expression; it does not demonstrate synapse formation, and the model is best suited for studying early differentiation, neurite dynamics, and pre‐synaptic or pre‐network aspects of neuronal biology, rather than synaptic transmission or network‐level function. The observed improvements should be interpreted in the context of enhanced morphological differentiation and stabilization of neuron‐like characteristics, rather than as the establishment of fully functional neuronal circuits.

In addition, absolute neurite length values may vary considerably across studies due to differences in experimental conditions (e.g., substrate, differentiation protocol, and analysis methods); therefore, these measurements should be interpreted primarily in terms of relative differences within the same experimental system (Dwane et al. 2013; Kaya et al. 2024; Freire et al. 2002; Kang and Yao 2022).

Moreover, claims regarding cost‐effectiveness should be interpreted cautiously, as Matrigel is expensive and exhibits batch‐to‐batch variability (Kaya et al. 2024; Bilginer Kartal and Arslan Yildiz 2024). However, in the context of this study, the reference to cost‐effectiveness was based on the use of a low LrECM concentration (1:100 coating), which substantially reduces reagent consumption and allows a large number of experiments to be performed with a single vial of matrix. This not only minimizes overall material costs but also mitigates issues related to batch‐to‐batch variability, a known limitation of Matrigel. Notably, such dilute concentrations have been reported to more closely approximate the mechanical stiffness of neuronal tissues, thereby providing a more physiologically relevant microenvironment for differentiation toward a neuronal phenotype (Traldi et al. 2023; Kang et al. 2017; Teixeira et al. 2009). In addition, the shorter duration of the protocol (4 days) compared to conventional RA‐only differentiation (7 days) contributes to a more time‐ and resource‐efficient workflow, even though absolute cost comparisons were not performed.

Consistent with previous reports, ECM components contribute to phenotypic maintenance by providing structural support and biochemical cues that influence adhesion, differentiation, and survival (Dwane et al. 2013; Kaya et al. 2024; Freire et al. 2002; Kang and Yao 2022; Li and Chau 2010; Mukherjee et al. 2020; Sahab Negah et al. 2018). In this study, the RA + LrECM condition preserved β3‐tubulin and NeuN expression over time and showed a modest, non‐significant increase in acetylcholinesterase (AChE) activity after 9 days. While this trend might suggest an early modulation of cholinergic enzyme activity, these results should be interpreted with caution. Further investigation, including the expression of specific markers such as choline acetyltransferase (ChAT) or vesicular acetylcholine transporter (VAChT), as well as functional assays like acetylcholine release, would be required to establish a more robust cholinergic phenotype. These results indicate that this approach can extend the duration of RA‐induced differentiation rather than producing a more mature or faster neuronal‐like phenotype.

In addition to the limitations previously discussed in this study, further topics should be considered. First, the LrECM used is a complex substrate containing multiple components, and therefore, the observed effects cannot be attributed exclusively to laminin. More defined ECM systems—such as purified laminin or synthetic matrices—offer great mechanistic clarity and reproducibility (Dwane et al. 2013; Kaya et al. 2024; Kovalevich et al. 2021; Leuenberger et al. 2026). Second, additional synaptic and neurotransmitter‐related markers would be valuable to further define the maturity and specificity of the differentiated phenotype. Third, β3‐tubulin expression was assessed by Western blotting without complementary immunocytochemical validation. Fourth, the distinction between N‐type and S‐type SH‐SY5Y cells was not assessed at the single‐cell level, which may contribute to heterogeneity within the differentiated population. Finally, functional assays such as calcium imaging or electrophysiological recordings were not performed. Therefore, the observed phenotype should be interpreted as neuron‐like rather than fully mature neurons.

In summary, combining RA with LrECM represents an incremental adjustment of existing SH‐SY5Y differentiation protocols. This approach can provide a more stable environment for maintaining neuron‐like characteristics at later time points. It may serve as a practical model for studies focusing on neuronal maintenance and neurodegenerative mechanisms, including Alzheimer's disease (Bronfman et al. 1996; Ferreira et al. 2018).

## 5 Conclusion

Overall, our results demonstrate that incorporating LrECM proteins into the differentiation process of SH‐SY5Y cells enhances the acquisition and maintenance of neuron‐like morphological and molecular characteristics. This effect is likely mediated by interactions between cell adhesion molecules and ECM components, which provide a microenvironment that partially mimics aspects of the neural milieu. Importantly, the present model reflects an early neuronal‐like phenotype and does not fully recapitulate mature, synaptically connected neuronal networks. This protocol does not demonstrate synapse formation, and the model is best suited for studying early differentiation, neurite dynamics, and pre‐synaptic or pre‐network aspects of neuronal biology, rather than synaptic transmission or network‐level function. Nevertheless, further studies incorporating functional assays will be required to evaluate neuronal‐like activity and to determine the extent to which this model can support investigations of more advanced neuronal‐like properties.

The RA + LrECM approach is a useful and practical model for studies of early neuronal‐like differentiation, neurite dynamics, and the stabilization of neuron‐like features over time. Therefore, this study contributes to refining SH‐SY5Y differentiation strategies by improving phenotypic stability under optimized culture conditions. Nevertheless, further studies incorporating functional assays will be required to evaluate neuronal‐like activity and to determine the extent to which this model can support investigations of more advanced neuronal‐like properties.

## Author Contributions

**Marina Mantellatto Grigoli:** conceptualization, data curation, software, formal analysis, investigation, methodology, writing – original draft. **Bianca Cruz Pachane:** data curation, investigation, software, formal analysis, validation, writing – original draft. **Angelina Maria Fuzer:** conceptualization, data curation, supervision, writing – original draft. **Sabrina Dorta de Oliveira:** investigation, methodology, writing – original draft. **Ana Beatriz Aparecida Targas:** investigation, methodology, visualization. **Vanessa Alexandre‐Silva:** writing – original draft. **Heloisa Sobreiro Selistre‐de‐Araujo:** supervision, resources, visualization. **Patricia Regina Manzine:** supervision, writing – review and editing. **Marcia Regina Cominetti:** conceptualization, funding acquisition, project administration, software, supervision, resources, writing – review and editing.

## Funding

This work was funded by the São Paulo Research Foundation (FAPESP) through grants 2021/01906‐0 and 2023/08952‐2 (to MMG); 2019/05149‐9 and 2021/01983‐4 (to BCP); 2021/01863‐9, 2021/14673‐3 and 2024/23624‐4 (to MRC); Coordenação de Aperfeiçoamento de Pessoal de Nível Superior (CAPES), code 001; and Conselho Nacional de Desenvolvimento Científico e Tecnológico (CNPq, 441066/2023‐2 to MRC and 174872/2023‐2 to AMF). The funding agencies had no direct involvement in the study's design, conduct, analysis, or reporting.

## Ethics Statement

The authors state that all research conducted in this study adhered to the highest ethical standards, ensuring integrity in research practices and compliance with institutional and legal requirements.

## Supporting information


**Data S1:** ImageJ pipeline for neurite measurement.
**Data S2:** ImageJ pipeline for fluorescence quantification.
**Figure S1:** Integer Western blotting membranes and cell confluence.

## Data Availability

The data sets generated during and/or analyzed during the current study are available from the corresponding author upon reasonable request. This study does not contain artificial intelligence‐generated data.

## References

[dvg70066-bib-0001] Agholme, L. , T. Lindstrom , K. Kagedal , J. Marcusson , and M. Hallbeck . 2010. “An in Vitro Model for Neuroscience: Differentiation of SH‐SY5Y Cells Into Cells With Morphological and Biochemical Characteristics of Mature Neurons.” Journal of Alzheimer's Disease 20, no. 4: 1069–1082. 10.3233/JAD-2010-091363 20413890

[dvg70066-bib-0002] Bilginer Kartal, R. , and A. Arslan Yildiz . 2024. “Exploring Neuronal Differentiation Profiles in SH‐SY5Y Cells Through Magnetic Levitation Analysis.” ACS Omega 9, no. 13: 14955–14962. 38585102 10.1021/acsomega.3c08962 PMC10993277

[dvg70066-bib-0003] Bronfman, F. C. , C. Soto , L. Tapia , V. Tapia , and N. C. Inestrosa . 1996. “Extracellular Matrix Regulates the Amount of the Beta‐Amyloid Precursor Protein and Its Amyloidogenic Fragments.” Journal of Cellular Physiology 166, no. 2: 360–369. 8591996 10.1002/(SICI)1097-4652(199602)166:2<360::AID-JCP14>3.0.CO;2-F

[dvg70066-bib-0004] Chize, C. M. , D. G. Vivas , K. Menezes , et al. 2025. “A Laminin‐Based Therapy for Dogs With Chronic Spinal Cord Injury: Promising Results of a Longitudinal Trial.” Frontiers in Veterinary Science 12: 1592687. 40881640 10.3389/fvets.2025.1592687 PMC12380836

[dvg70066-bib-0005] de Medeiros, L. M. , M. A. De Bastiani , E. P. Rico , et al. 2019. “Cholinergic Differentiation of Human Neuroblastoma SH‐SY5Y Cell Line and Its Potential Use as an in Vitro Model for Alzheimer's Disease Studies.” Molecular Neurobiology 56, no. 11: 7355–7367. 31037648 10.1007/s12035-019-1605-3

[dvg70066-bib-0006] Dwane, S. , E. Durack , and P. A. Kiely . 2013. “Optimising Parameters for the Differentiation of SH‐SY5Y Cells to Study Cell Adhesion and Cell Migration.” BMC Research Notes 6: 366. 24025096 10.1186/1756-0500-6-366 PMC3847106

[dvg70066-bib-0007] Feles, S. , C. Overath , S. Reichardt , et al. 2022. “Streamlining Culture Conditions for the Neuroblastoma Cell Line SH‐SY5Y: A Prerequisite for Functional Studies.” Methods and Protocols 5, no. 4: 58. 35893584 10.3390/mps5040058 PMC9326679

[dvg70066-bib-0008] Ferreira, L. S. S. , C. S. Fernandes , M. N. N. Vieira , and F. G. De Felice . 2018. “Insulin Resistance in Alzheimer's Disease.” Frontiers in Neuroscience 12: 830. 30542257 10.3389/fnins.2018.00830 PMC6277874

[dvg70066-bib-0009] Freire, E. , F. C. Gomes , R. Linden , V. M. Neto , and T. Coelho‐Sampaio . 2002. “Structure of Laminin Substrate Modulates Cellular Signaling for Neuritogenesis.” Journal of Cell Science 115, no. Pt 24: 4867–4876. 12432074 10.1242/jcs.00173

[dvg70066-bib-0010] Jahn, K. , C. Wieltsch , N. Blumer , et al. 2017. “A Cell Culture Model for Investigation of Synapse Influenceability: Epigenetics, Expression and Function of Gene Targets Important for Synapse Formation and Preservation in SH‐SY5Y Neuroblastoma Cells Differentiated by Retinoic Acid.” Journal of Neural Transmission 124, no. 11: 1341–1367. 28887651 10.1007/s00702-017-1769-9

[dvg70066-bib-0011] Kang, M. , and Y. Yao . 2022. “Laminin Regulates Oligodendrocyte Development and Myelination.” Glia 70, no. 3: 414–429. 34773273 10.1002/glia.24117 PMC8817735

[dvg70066-bib-0012] Kang, P. H. , S. Kumar , and D. V. J. Schaffer . 2017. “Novel Biomaterials to Study Neural Stem Cell Mechanobiology and Improve Cell‐Replacement Therapies.” Current Opinion in Biomedical Engineering 4: 13–20. 29399646 10.1016/j.cobme.2017.09.005 PMC5791915

[dvg70066-bib-0013] Kaya, Z. B. , V. Santiago‐Padilla , M. Lim , S. L. Boschen , P. Atilla , and P. J. McLean . 2024. “Optimizing SH‐SY5Y Cell Culture: Exploring the Beneficial Effects of an Alternative Media Supplement on Cell Proliferation and Viability.” Scientific Reports 14, no. 1: 4775. 38413790 10.1038/s41598-024-55516-5 PMC10899233

[dvg70066-bib-0014] Kovalevich, J. , M. Santerre , and D. Langford . 2021. “Considerations for the Use of SH‐SY5Y Neuroblastoma Cells in Neurobiology.” Methods in Molecular Biology 2311: 9–23. 34033074 10.1007/978-1-0716-1437-2_2

[dvg70066-bib-0015] Leuenberger, J. , G. Ott , T. Nevian , B. Zuber , and I. Rostami . 2026. “Differentiated SH‐SY5Y Cells Exhibit Neuronal Features but Lack Synaptic Maturity.” Cell Death Discovery 12: 1–10. 10.1038/s41420-026-03094-y PMC13369904 41980924

[dvg70066-bib-0016] Li, Q. , and Y. Chau . 2010. “Neural Differentiation Directed by Self‐Assembling Peptide Scaffolds Presenting Laminin‐Derived Epitopes.” Journal of Biomedical Materials Research. Part A 94, no. 3: 688–699. 20730926 10.1002/jbm.a.32707

[dvg70066-bib-0017] Menezes, K. , M. A. B. Lima , D. R. Xerez , et al. 2024. “Return of Voluntary Motor Contraction After Complete Spinal Cord Injury: A Pilot Human Study on POLYLAMININ.” 10.1101/2024.02.19.24301010.

[dvg70066-bib-0018] Mesquita, F. C. P. , E. S. Leite , J. Morrissey , C. Freitas , T. Coelho‐Sampaio , and C. Hochman‐Mendez . 2022. “Polymerized Laminin‐521: A Feasible Substrate for Expanding Induced Pluripotent Stem Cells at a Low Protein Concentration.” Cells 11, no. 24: 3955. 36552719 10.3390/cells11243955 PMC9777247

[dvg70066-bib-0019] Minegishi, T. , R. F. Kastian , and N. Inagaki . 2023. “Mechanical Regulation of Synapse Formation and Plasticity.” Seminars in Cell & Developmental Biology 140: 82–89. 35659473 10.1016/j.semcdb.2022.05.017

[dvg70066-bib-0020] Mukherjee, C. , S. Saleem , S. Das , S. C. Biswas , and D. Bhattacharyya . 2020. “Human Placental Laminin: Role in Neuronal Differentiation, Cell Adhesion and Proliferation.” Journal of Biosciences 45: 93. 32713856

[dvg70066-bib-0021] Pahlman, S. , A. I. Ruusala , L. Abrahamsson , M. E. Mattsson , and T. Esscher . 1984. “Retinoic Acid‐Induced Differentiation of Cultured Human Neuroblastoma Cells: A Comparison With Phorbolester‐Induced Differentiation.” Cell Differentiation 14, no. 2: 135–144. 6467378 10.1016/0045-6039(84)90038-1

[dvg70066-bib-0022] Pemberton, K. , B. Mersman , and F. Xu . 2018. “Using ImageJ to Assess Neurite Outgrowth in Mammalian Cell Cultures: Research Data Quantification Exercises in Undergraduate Neuroscience Lab.” Journal of Undergraduate Neuroscience Education 16, no. 2: A186–A194. 30057501 PMC6057772

[dvg70066-bib-0023] Powell, S. K. , and H. K. Kleinman . 1997. “Neuronal Laminins and Their Cellular Receptors.” International Journal of Biochemistry & Cell Biology 29, no. 3: 401–414. 9202420 10.1016/s1357-2725(96)00110-0

[dvg70066-bib-0024] Sahab Negah, S. , A. Khooei , F. Samini , and A. Gorji . 2018. “Laminin‐Derived Ile‐Lys‐Val‐Ala‐Val: A Promising Bioactive Peptide in Neural Tissue Engineering in Traumatic Brain Injury.” Cell and Tissue Research 371, no. 2: 223–236. 29082446 10.1007/s00441-017-2717-6

[dvg70066-bib-0025] Schindelin, J. , I. Arganda‐Carreras , E. Frise , et al. 2012. “Fiji: An Open‐Source Platform for Biological‐Image Analysis.” Nature Methods 9, no. 7: 676–682. 22743772 10.1038/nmeth.2019 PMC3855844

[dvg70066-bib-0026] Shipley, M. M. , C. A. Mangold , and M. L. Szpara . 2016. “Differentiation of the SH‐SY5Y Human Neuroblastoma Cell Line.” Journal of Visualized Experiments 108: 53193. 10.3791/53193 PMC4828168 26967710

[dvg70066-bib-0027] Strother, L. , G. B. Miles , A. R. Holiday , Y. Cheng , and G. H. Doherty . 2021. “Long‐Term Culture of SH‐SY5Y Neuroblastoma Cells in the Absence of Neurotrophins: A Novel Model of Neuronal Ageing.” Journal of Neuroscience Methods 362: 109301. 34343572 10.1016/j.jneumeth.2021.109301 PMC8434422

[dvg70066-bib-0028] Teixeira, A. I. , S. Ilkhanizadeh , J. A. Wigenius , J. K. Duckworth , O. Inganäs , and O. Hermanson . 2009. “The Promotion of Neuronal Maturation on Soft Substrates.” Biomaterials 30, no. 27: 4567–4572. 19500834 10.1016/j.biomaterials.2009.05.013

[dvg70066-bib-0029] Traldi, C. , V. Chiappini , G. Menduti , C. Tonda‐Turo , and M. Boido . 2023. “Advanced Materials and Biofabrication Technologies to Design in Vitro Functional Central Nervous System Models.” Frontiers in Medical Engineering 1: 1270943.

[dvg70066-bib-0030] Waetzig, V. , W. Haeusgen , C. Andres , et al. 2019. “Retinoic Acid‐Induced Survival Effects in SH‐SY5Y Neuroblastoma Cells.” Journal of Cellular Biochemistry 120, no. 4: 5974–5986. 30320919 10.1002/jcb.27885

[dvg70066-bib-0031] Xicoy, H. , B. Wieringa , and G. J. Martens . 2017. “The SH‐SY5Y Cell Line in Parkinson's Disease Research: A Systematic Review.” Molecular Neurodegeneration 12, no. 1: 10. 28118852 10.1186/s13024-017-0149-0 PMC5259880

