# Supplementary material for: An Efficient Method for Neuron‐Like Differentiation of SH‐SY5Y Neuroblastoma Cells Using Retinoic Acid and Laminin‐Rich Extracellular Matrix
Source: Genesis. 2026 Aug 2;64(4):e70066. doi: 10.1002/dvg.70066 (PMC13429946; doi:10.1002/dvg.70066)
Supplement: Supplementary file 1 — Data S1: ImageJ pipeline for neurite measurement. Data S2: ImageJ pipeline for fluorescence quantification. Figure S1: Integer Western blotting membranes and cell confluence. [file DVG-64-e70066-s001.docx]

**Supplementary Information**

**Supplementary Information 1** -ImageJ pipeline for neurite measurement

*> setScale*

*> open(StackedFile.tif);*

*> selectImage("StackedFile.tif");*

*> run("Stack to Images");*

*> selectImage("Image1");*

*> run("Find Maxima...", "prominence=50 light output=[Segmented Particles]");*

*//”SegmentedImage1”;*

*> selectImage("Image1");*

*> setAutoThreshold("Huang no-reset");*

*//run("Threshold...");*

*//setThreshold(0, 101);*

*> setOption("BlackBackground", true);*

*> run("Convert to Mask");*

*> imageCalculator("AND create", "SegmentedImage1","Image1");*

*> selectImage("Result of SegmentedImage1");*

*> run("Skeletonize");*

*> run("Analyze Skeleton (2D/3D)", "prune=[shortest branch] calculate show display");*

*> saveAs("Results".csv").*

**Supplementary Information 2 -**  ImageJ pipeline for fluorescence quantification

*> setScale*

*> open(StackedFile.tif);*

*> selectImage("StackedFile.tif");*

*> run("Split Channels");*

*> selectImage("C1-File");*

*> close();*

*> selectImage("C2-File");*

*> close();*

*> selectImage("C3-File");*

*> run("Duplicate...", "use");*

*> setMinAndMax(15, 1018);*

*> setAutoThreshold("Huang dark no-reset");*

*//run("Threshold...");*

*> setThreshold(175, 65535, "raw");*

*> run("Smooth");*

*> run("Convert to Mask");*

*> selectImage("C3-File");*

*> run("Enhance Contrast...", "saturated=0.35 normalize equalize");*

*> run("Subtract...", "value=14000");*

*> run("Find Maxima...", "prominence=20000 output=[Segmented Particles]");*

*> imageCalculator("AND create", "C3-File Segmented");*

*> selectImage("Result of C3-File Segmented");*

*> run("Analyze Particles...", "size=15.00-Infinity show=Overlay display summarize add");*

*> saveAs("Results".csv").*

**Supplementary Figure 1** - Integer Western blotting membranes and cell confluence

*
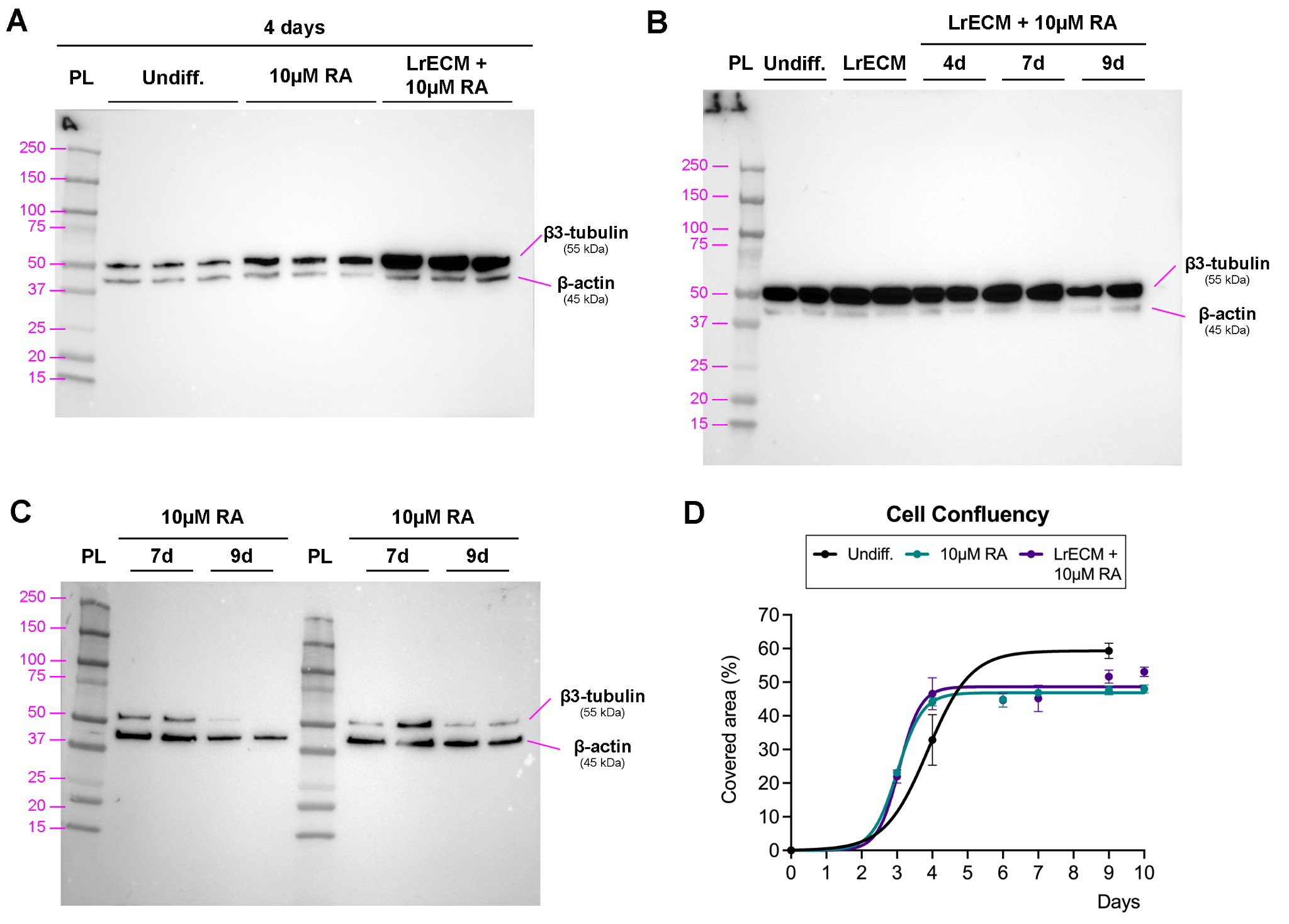
*

(A-C) Integer Western blotting membrane for β3-tubulin (55 kDa) and β-actin (45 kDa) detection of SH-SY5Y lysates from undifferentiated cells (Undiff.) and cells differentiated with retinoic acid (RA) and laminin-rich extracellular matrix (LrECM + 10 µM RA), with molecular weights described in magenta. (D) Cell confluency graph of covered area (%) per day of undifferentiated cells (black), cells treated with 10 µM RA (cyan) and co-treatment of LrECM and 10 µM RA (purple) (mean ± SD).
